# Subcutaneous Fat Necrosis of the Newborn After Whole-Body Cooling for Birth Asphyxia

**DOI:** 10.7759/cureus.34521

**Published:** 2023-02-01

**Authors:** Yamen Elmoghanni, Asma Aldhafer, Mona Khalaf, Funda Cipe, Gangaiah Komala, Tausif Saleem, Sinan Yavuz

**Affiliations:** 1 Pediatrics, Al Qassimi Women's and Children's Hospital, Sharjah, ARE; 2 Pathology, Al Qassimi Hospital, Sharjah, ARE; 3 Histopathology, Sheikh Khalifa Medical City, Abu Dhabi, ARE

**Keywords:** birth asphyxia, therapeutic hypothermia, subcutaneous fat necrosis, newborn rash, hypercalcemia, term neonates

## Abstract

Therapeutic hypothermia (TH) is a challenging treatment for a neonate who suffers from hypoxic-ischemic encephalopathy. It has been shown to improve neurodevelopmental outcomes and survival in infants with moderate-to-severe hypoxic-ischemic encephalopathy. However, it has severe adverse effects such as subcutaneous fat necrosis (SCFN). SCFN is a rare disorder that affects term neonates. It is a self-limited disorder but can have severe complications such as hypercalcemia, hypoglycemia, metastatic calcifications, and thrombocytopenia. In this case report, we present a term newborn who developed SCFN after whole-body cooling.

## Introduction

Perinatal asphyxia is a decrease of blood flow or gas exchange disturbance to or from the fetus immediately before, during, or after delivery. It can affect different parts of the body, such as the brain, heart, liver, kidney, and skin [1]. Whole-body cooling is the standard treatment for moderate-to-severe hypoxic-ischemic encephalopathy. The common side effects of therapeutic hypothermia (TH) are bradycardia, hypotension, and prolonged coagulation profiles [2]. Subcutaneous fat necrosis (SCFN) is a rare side effect of TH, which is estimated to be around 1% [3-5]. The symptoms are indurated erythematous nodules and plaques on the back, arms, buttocks, thighs, and cheeks [6]. Pain scarring and hypercalcemia are possible complications that can be developed even after weeks of SCFN, and they may need treatment if symptomatic [7].

Here, we present a newborn who developed SCFN after whole-body cooling for birth asphyxia due to meconium aspiration syndrome.

## Case presentation

A full-term 39-week and five-day-old baby boy was born to a 33-year-old mother via an emergency cesarean section for gravida 2 para 2 mother thick meconium-stained liquor. The mother’s pregnancy had been uncomplicated. At delivery, the neonate was cyanotic, floppy, and apneic. His initial appearance, pulse, grimace, activity, and respiration (APGAR) scores were one at five minutes and six at 10 minutes. The umbilical cord blood gas indicated a severe metabolic acidosis, and the neonate was intubated in the delivery room and received fluid resuscitation. His initial capillary blood gas (CBG) was pH: 7.03, PCO_2_: 55, HCO_3_: 14.2, and BE: 17. His blood sugar was critically low (unrecordable), and he needed a dextrose 10% bolus to correct it. Initial blood tests and liver and kidney function tests were abnormal, and albumin and total protein were low. The calcium level was 2.32 mg/dl. White blood cell counts were normal and platelet was low, that is, 74,000/mcL. The C-reactive protein (CRP) was 10 mg/L (range 0-6 mg/L). Antibiotics penicillin and gentamicin were started as per the protocol. An initial echocardiogram (ECHO) showed severe pulmonary hypertension.

At eight hours after birth, the baby developed periodic breathing, abnormal tones in his upper and lower limbs with smacking, Moro incompleted, lethargic, and cerebral function monitor tracing was abnormal, for which he was loaded with phenobarbitone, and TH was started for 72 hours (Sarnat stage 2). A cooling mattress was set under the baby to maintain a target temperature of 33.5°C for 72 hours. Then, he was gradually rewarmed until a temperature of 36.5°C was reached.

During the cooling process, the baby began to have hypotension and required inotropic support, which weaned gradually after 48 hours. At 72 hours of life, the baby’s neurologic status had markedly improved; he became alert and responded to stimuli, and the results of his neurologic examination were normal.

**Figure 1:**
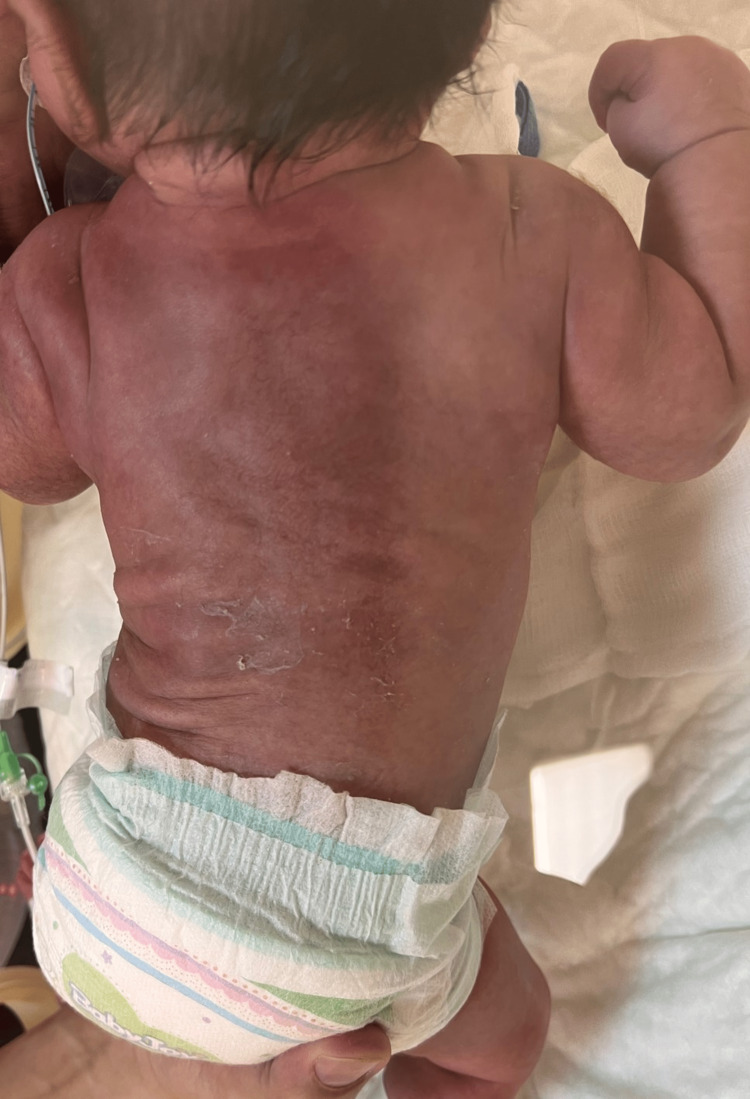
Skin lesions due to subcutaneous fat necrosis in the patient; erythematous to violaceous tender, indurated plaque swelling on the back and shoulders.

On day four, after the completion of TH, the baby was noticed to have erythematous to violaceous tender, indurated plaque swelling on the back and the shoulders (Figure 1).

**Figure 2:**
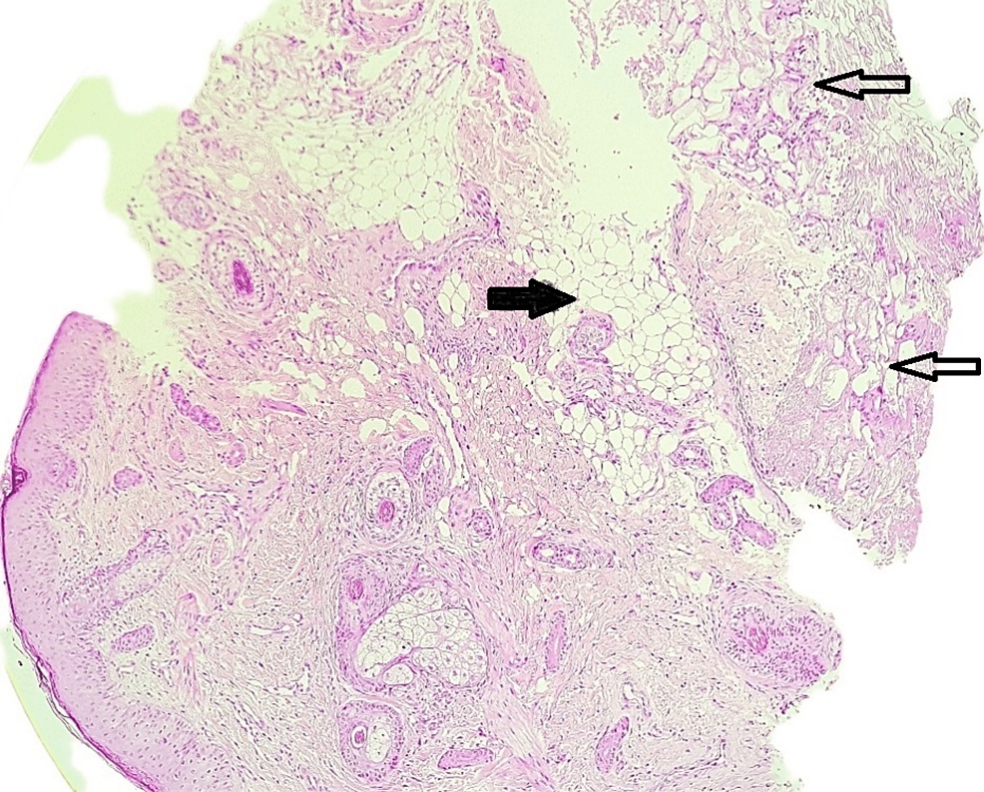
Skin biopsy revealed the superficial fat zone (black arrow) has distinct morphology compared to the deeper zone (white arrows) under low power.

**Figure 3:**
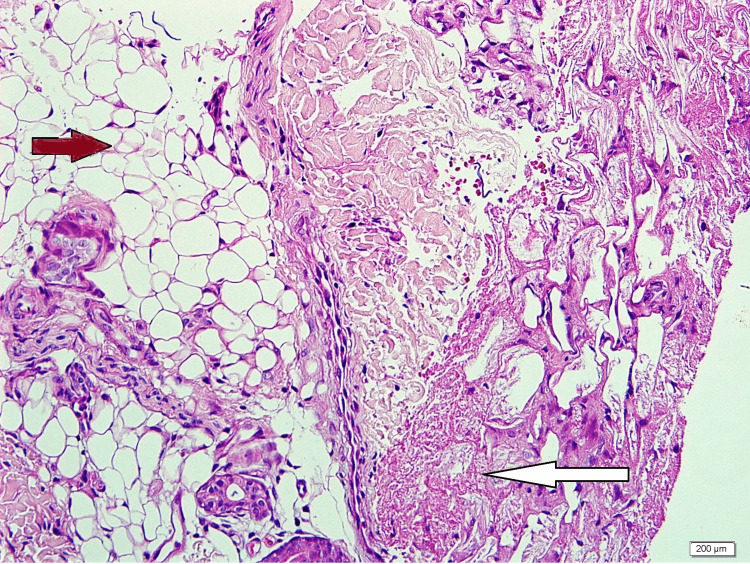
The skin biopsy revealed that the higher power focusing on subcutaneous fat shows superficial fat lobules intact with well-preserved adipocytes (brown arrow). The deeper fat shows collapsed morphology with loss of distinct cell borders and necrosis (white arrow). No obvious inflammatory infiltrate. These features indicate lobular panniculitis.

A skin biopsy was taken, which revealed a higher power focusing on subcutaneous fat, showing superficial fat lobules intact with well-preserved adipocytes. The deeper fat showed collapsed morphology with the loss of distinct cell borders and necrosis. No obvious inflammatory infiltrate was observed. These features indicated lobular panniculitis (Figures 2, 3).

The magnetic resonance imaging of the brain on day seven showed a normal study. The electroencephalogram reported a normal study, and the echocardiogram report was normal.

On day 14, the baby developed persistent hypercalcemia. Serum ionized calcium >1.75 mmol/l (normal range 1.05 to 1.37 mmol/l) associated with poor feedings was observed. The vitamin D supplement was stopped, and he was given a volume expansion of double maintenance followed by one shot of furosemide. The ionized calcium level reduced to 1.55 mmol/l; however, after 24 hours, it started to increase to 1.8 mmol/l. The endocrinologist advised to keep the baby hydrated and started on prednisolone once daily (1.5 mg/kg/day) with intravenous (IV) of furosemide 1 mg/kg every six hours with a low calcium diet in addition to IV fluids. His calcium level began to improve gradually over one week, with the skin lesion subsiding without any complications after six weeks. The repeated blood tests and renal ultrasound (US) were normal. His sucking and feeding process improved, and he was discharged in good condition.

## Discussion

The SCFN in neonates is self-limited panniculitis in term and post-term neonates with full recovery within weeks or months. Its symptoms are indurated erythematous nodules and plaques on the back, arms, buttocks, thighs, and cheeks [6]. The pathophysiology of SCFN remains unclear; however, under stress, tissue hypoxia with impaired perfusion leads to the crystallization of subcutaneous fat tissues, which results in tissue necrosis [8].

A punch biopsy is the gold-standard procedure to confirm the diagnosis. Histopathological findings are lobular panniculitis with adipose cell necrosis and dense inflammatory infiltrates involving lymphocytes, histiocytes, giant cells in granuloma formation, lipophages, and rare eosinophils [9]. Fine needle aspiration and touch preparation for spontaneously draining lesions are other options to confirm the diagnosis of SCFN. Even though they are rapid and less invasive, they lack sensitivity compared to skin biopsies [10,11]. International studies have shown improved mortality and morbidity of neonates with hypoxic-ischemic encephalopathy who underwent whole-body cooling without mentioning the adverse dermatological effect [2,12]. However, another control trial study has revealed that four of the 102 patients had developed dermatologic complications of TH, such as erythema, sclerema, cyanosis, and SCFN [2]. Moreover, one case series showed a possible association between TH and SCFN [13].

The most common risk factors of SCFN are hypothermia and birth hypoxia. Other less common risk factors are pre-eclampsia, maternal diabetes, cocaine abuse or use of calcium channel blockers during pregnancy, birth asphyxia, meconium, aspiration, hypoxemia, and hypoglycemia [14]. In the patients we studied, the most common predisposing factor was whole-body cooling due to the development of lesions on the back of the patient where the cooling mattress was kept.

Hypercalcemia is a rare serious complication of SCFN that can develop due to elevation in the prostaglandin activity, released calcium from necrotic fat, and raised macrophage secretion of 1,2-dihydroxy vitamin D [15]. It can be asymptomatic, but some of the patients require treatment. The signs and symptoms are lethargy, hypotonia, irritability, vomiting, polyuria, polydipsia, constipation, and dehydration [6]. It can be managed by low-calcium and vitamin D formula, adequate fluids, and furosemide or low-dose corticosteroids [16,17].

Another risk factor of SCFN is hypoglycemia, whose role is unknown. Gestational diabetes is an important cause of hypoglycemia. The SCFN occurs even in the absence of maternal diabetes [18,19]. Thrombocythemia occurs due to the sequestration of platelets in the subcutaneous nodules [20]; however, our patient has had it since admission. This condition normalized after a long time and required a platelet transfusion. The SCFN has been found to have a role in long-term thrombocytopenia [20].

## Conclusions

The SCFN is a benign, self-limited condition. Therapeutic cooling is one of the risk factors of SCFN, and neonates who require treatment with TH should monitor the skin regularly. However, it may develop complications such as hypoglycemia, thrombocytopenia, and especially hypercalcemia. It is very important to monitor the patient closely, especially in the case of hypercalcemia.
